# Keystone

**Published:** 1896-04

**Authors:** W. L. Rhoads

**Affiliations:** Secretary


Secretary.

### KEYSTONE VETERINARY MEDICAL ASSOCIATION.

THE March meeting of the Association was called to order by President John R. Hart, at 8 P.M., on the 10th inst. Coming so near the State meeting, the attendance was remarkably light; yet during the discussion on "Azoturia" many new and valuable ideas were advanced. Those present were Drs. John R. Hart, T. B. Rayner, Chas. Lintz, J. Johnston, Jas. T. McAnulty, H. J. McClellan, W. H. Hoskins, W. L. Rhoads, and F. S. Allen.

The Chairman of the Legislative Committee reported that, as instructed at the last meeting, they had formulated and mailed letters to everyone whom they thought needed more urging on the bill granting rank to the army veterinarians. After this report Dr. Lintz offered a resolution extending a vote of thanks to Dr. Hoskins as Chairman of the Legislative Committee for the great amount of work done and the very creditable methods pursued.

A communication from Dr. H. B. Felton, containing his resignation from active membership in the Keystone Veterinary Medical Association, was now read. The resignation was immediately acted upon and accepted.

Dr. F. S. Allen reported three cases of azoturia, one subject having two attacks within five weeks, the recovery from the second attack being the most rapid and complete. The first was a black gelding, five years old, 16½ hands high. Given purgatives and diaphoretics; after second day began to improve; the muscular action of the near hind-limb had become inert; this was treated with liniment and friction and eventually a blister. In the treatment of the second case he used fluid-extract buchu, fluid-extract juniper, and acetate potash instead of ammonia; had very satisfactory results. After an interval of about one month this horse was taken down again; he gave him the same remedies as before in conjunction with a half-pound of bicarbonate soda every six hours. The results from this line of treatment were almost phenomenal, the recovery being made in a much shorter space of time than with the earlier treatment. Dr. Johnston said that in many such cases the characteristic urine was not albuminous, but contained an excess of salts. Dr. Allen thought the weather had a great influence, and said he found it mostly where they made a practice of feeding very heavy oats, thus saturating the system with proteids. Dr. Hoskins spoke of noting, in several cases, that the urine, at first was straw color, and became darker and more characteristic each time it was removed. In Germany they use ice in bags of bran as a cold pack along the spine, and claim good results. This treatment has proved beneficial in a case reported by one of our local practitioners. Dr. Lintz cited two cases occurring in June, one travelling over twenty miles, and made good recovery. Dr. Johnston spoke of a case in a two-year-old trotting-bred colt; attack came on after very fast work on a very cold day; his symptoms came on gradually, but he made good recovery in five weeks.

The Secretary announced the receipt of an application from Dr. F. S. Allen for membership, which will be acted upon at the April meeting.

The Society adjourned to reconvene April 14, 1896.

W. L. Rhoads,
Secretary.

